# Advancing precision medicine in axial spondyloarthritis: insights from multi-omics approaches

**DOI:** 10.3389/fmed.2025.1715420

**Published:** 2025-11-10

**Authors:** Yuanpiao Ni, Quanbo Zhang, Xin Wu, Huji Xu, Yufeng Qing

**Affiliations:** 1 Research Center of Hyperuricemia and Gout, Affiliated Hospital of North Sichuan Medical College, Nanchong, Sichuan, China; 2 Department of Rheumatology and Immunology, Affiliated Hospital of North Sichuan Medical College, Nanchong, Sichuan, China; 3 Department of Rheumatology and Immunology, Mianyang Central Hospital, Mianyang, Sichuan, China; 4 Department of Geriatrics, Affiliated Hospital of North Sichuan Medical College, Nanchong, China; 5 Department of Rheumatology and Immunology, Shanghai Changzheng Hospital, Naval Medical University, Shanghai, China

**Keywords:** axial spondyloarthritis, multi-omics integration, precision medicine, artificial intelligence, disease biomarker

## Abstract

Axial Spondyloarthritis (axSpA) is a chronic inflammatory disease influenced by genetic, immune, metabolic, and environmental factors, significantly impacting patients’ quality of life. Recent advancements in multi-omics technologies—such as genomics, transcriptomics, proteomics, and metabolomics—provide new insights into axSpA pathogenesis and precision medicine. These technologies reveal genetic susceptibility, immune responses, and metabolic alterations, uncovering potential biomarkers and therapeutic targets. This review explores multi-omics applications in understanding axSpA mechanisms, developing targeted therapies, and advancing precision diagnostics. It also addresses challenges in data integration and highlights the role of artificial intelligence (AI) in enhancing analysis precision and constructing dynamic disease networks. Combining AI with multi-omics could revolutionize diagnosis, personalized treatment, and clinical translation for axSpA, driving the future of precision medicine.

## 1 Introduction

AxSpA is a chronic, progressive inflammatory disease primarily affecting the spine and sacroiliac joints (1). It often leads to persistent pain, stiffness, and limited mobility, potentially resulting in spinal deformities and loss of function (2). The pathogenesis of axSpA is complex, involving the interplay of genetic predisposition, immune system abnormalities, and environmental factors (3–5). Although significant progress has been made in axSpA treatment, existing approaches still face notable limitations in early diagnosis, personalized treatment, and long-term management, hindering the realization of precision medicine (6). In particular, the incomplete understanding of axSpA’s pathological mechanisms and the atypical nature of its early symptoms lead many patients to miss the optimal window for treatment, resulting in significant variability in therapeutic outcomes (7).

In recent years, the rapid advancement of high-throughput omics technologies, including genomics, transcriptomics, proteomics, and metabolomics, has provided new opportunities for axSpA research (8). These technologies enable researchers to explore the molecular mechanisms of axSpA from multiple dimensions, identify potential biomarkers, and support targeted and personalized therapies. For instance, genomic studies have elucidated genetic risk factors like the HLA-B27 gene, while transcriptomic analyses have revealed immune-related dysregulation in axSpA (9). However, single-omics approaches struggle to fully capture the complexity of the disease, and the challenges of data heterogeneity and integration limit their widespread clinical application.

In contrast to previous reviews, which primarily focus on individual omics layers, this review emphasizes the integrated application of multi-omics approaches, which offer a more holistic view of axSpA pathogenesis. By combining data across genomics, transcriptomics, proteomics, metabolomics, and microbiomics, we can better understand the disease’s complexity and identify patient subtypes, thereby laying the foundation for precision medicine in axSpA (10). However, processing vast amounts of omics data and uncovering underlying patterns remains a significant challenge in current research (11).

To address these challenges, AI has emerged as a transformative tool in the analysis and integration of multi-omics data. Machine learning and deep learning methods can uncover complex patterns and associations within large datasets, significantly improving the accuracy and efficiency of analyses (12). AI can automatically detect complex patterns within the data, enhancing the accuracy and efficiency of analyses, and offering new solutions for early diagnosis, disease prediction, and personalized treatment of axSpA. The integration of AI with multi-omics technologies not only aids in uncovering the pathophysiological mechanisms of axSpA but also facilitates clinical translation by constructing disease networks and identifying biomarkers for precision medicine.

This review provides a comprehensive examination of the current state of multi-omics research in axSpA, with a particular focus on how AI can facilitate the integration of omics data to enhance diagnostic and therapeutic precision. We also highlight the ongoing challenges and future prospects of integrating these technologies to drive the realization of personalized treatment strategies for axSpA, ultimately improving patient outcomes and quality of life.

## 2 Multi-omics research process in axial spondyloarthritis

**Figure 1:**
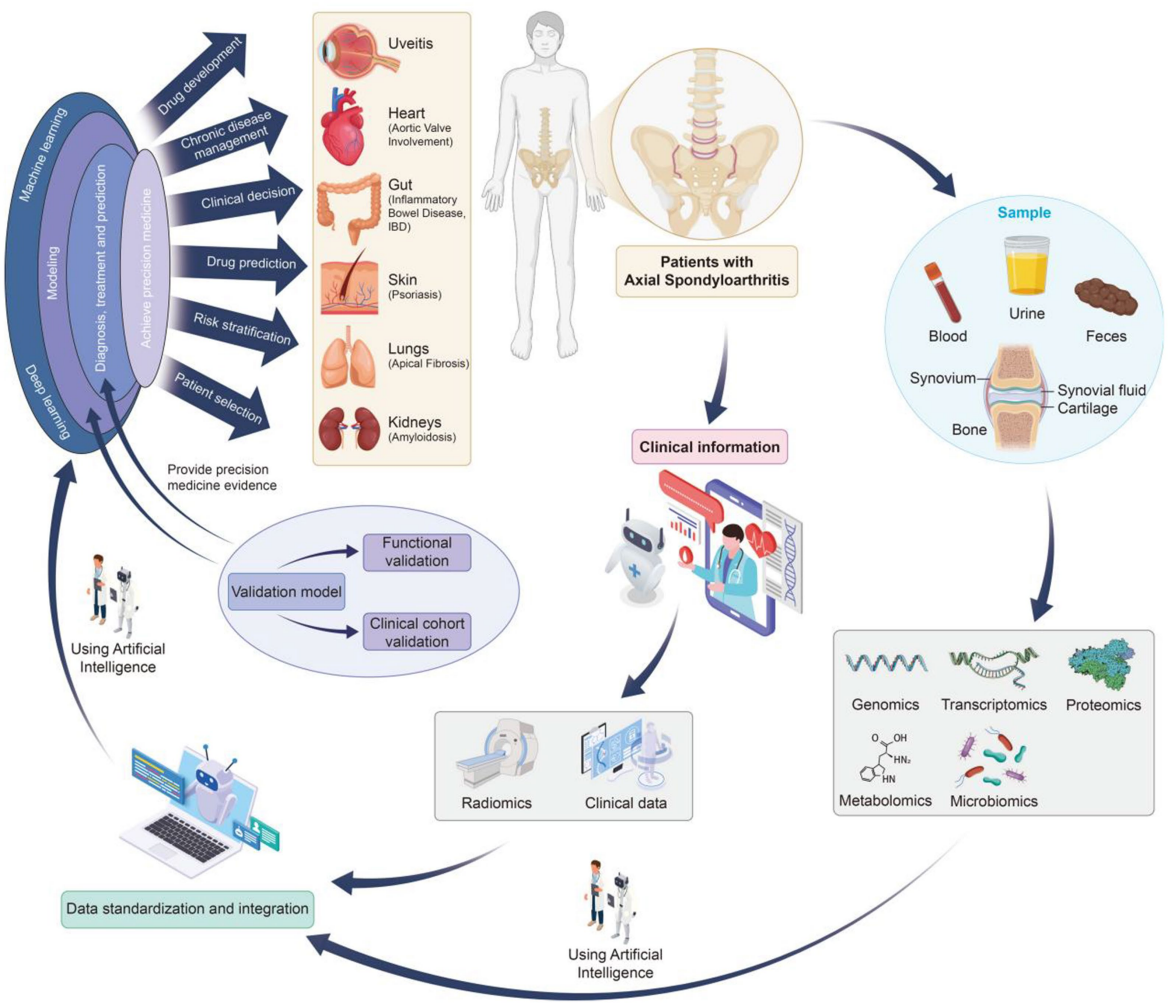
Multi-omics research process in axial spondyloarthritis.

The integration of multi-omics technologies provides a powerful approach for elucidating disease mechanisms, identifying reliable biomarkers, and advancing precision medicine in axSpA. Figure 1 illustrates a comprehensive multi-omics research workflow specifically tailored for axSpA.

### 2.1 Sample collection and preparation

The research process begins with the systematic collection of high-quality biological samples from axSpA patients and appropriate controls (13). These include blood, urine, feces, synovial fluid, cartilage, synovium, and bone tissue, representing the diverse tissues involved in axSpA-related inflammation and structural changes, particularly in the sacroiliac joints and axial skeleton.

As illustrated in Figure 1, while axSpA primarily affects the axial joints, especially the spine and sacroiliac joints, it may also involve peripheral joints and lead to extra-articular manifestations in organs such as the gut, eyes, skin, lungs, heart, and kidneys. This highlights the importance of comprehensive and multi-source biological sampling to capture the systemic nature of the disease.

Simultaneously, detailed clinical information—including disease duration, symptom profile, imaging findings, treatment history, and associated comorbidities—is collected. This clinical context is essential for correlating molecular findings with phenotypic presentations and enhancing the biological interpretation of omics-derived insights.

### 2.2 Omics data generation

Advanced high-throughput platforms are employed to generate multi-layered omics data from collected samples, encompassing:

Genomics (e.g., whole genome/exome sequencing): Identifies genetic variants and risk alleles associated with axSpA predisposition (9).

Transcriptomics (e.g., RNA-Seq): Profiles gene expression signatures and identifies dysregulated transcriptional networks in axSpA-affected tissues (14).

Proteomics (e.g., mass spectrometry): Quantifies protein abundance and post-translational modifications, elucidating protein-level alterations in disease progression (15).

Metabolomics (e.g., MS, NMR): Captures metabolic dysregulations and immune-metabolic interactions involved in axSpA pathogenesis (16).

Microbiomics: Investigates alterations in gut and mucosal microbiota, which are increasingly recognized as contributors to axSpA development (17, 18).

These diverse omics layers provide a multidimensional molecular atlas of axSpA, enabling comprehensive disease modeling.

### 2.3 Data preprocessing and quality control

Given the complexity and heterogeneity of omics datasets, rigorous data preprocessing is essential (19):

Data cleaning: Eliminates noise, contaminants, and low-confidence features.

Normalization: Adjusts for batch effects and technical variability, ensuring cross-sample comparability.

Missing data imputation: Employs statistical methods to handle incomplete entries, preserving dataset integrity.

These quality control measures are critical to ensure analytical reliability and reproducibility.

### 2.4 Multi-omics data integration and analysis

The integration of multi-omics datasets is essential for understanding the complexity of axSpA. By combining genomics, transcriptomics, proteomics, metabolomics, and microbiomics data, researchers can reveal systemic interactions driving axSpA pathophysiology. However, discrepancies often arise between different omics layers, such as genetic findings not fully aligning with transcriptomic or proteomic data. For instance, genetic risk factors identified in genomic studies may not always correlate directly with changes in gene expression or protein abundance, creating inconsistencies across datasets. These discrepancies are influenced by factors such as sample types, disease stages, or technical limitations in omics platforms.

To address these challenges, systematic data integration and cross-validation across omics layers are crucial. AI, particularly machine learning and deep learning techniques, aids in resolving these discrepancies by detecting hidden patterns across datasets, allowing for the harmonization of findings. This enables the identification of reliable biomarkers, disease subtypes, and therapeutic targets.

Despite the progress, challenges remain in ensuring the robustness and clinical applicability of multi-omics findings. Cross-validation using independent datasets is essential to confirm the reliability of biomarkers and therapeutic targets. Additionally, the development of standardized methods for integrating omics data and addressing data heterogeneity will improve the accuracy of multi-omics studies.

This systems-level approach provides a holistic view of axSpA, highlighting the critical role AI will continue to play in improving data integration, resolving inconsistencies, and facilitating the translation of omics insights into personalized treatment strategies. As discussed in Section 2.5, AI’s transformative role in data analysis further enhances the precision and applicability of these findings, leading to more effective diagnosis and treatment.

### 2.5 Artificial intelligence-assisted analysis

As illustrated in Figure 1, AI—particularly machine learning and deep learning—plays a pivotal role in the analysis and interpretation of multi-omics data (20). AI offers robust solutions for data integration, subtype discovery, and precision diagnostics, providing new opportunities for enhancing disease understanding and treatment strategies.

#### 2.5.1 Application of AI algorithms

Deep Learning (DL) and ensemble learning: Deep learning models, such as convolutional neural networks (CNNs) and recurrent neural networks (RNNs), are highly effective in processing complex, unstructured data like genomic sequences and transcriptomic data. These models excel at uncovering intricate patterns and identifying potential biomarkers within large datasets. In contrast, ensemble learning methods, such as random forests and XGBoost, are invaluable for integrating data from multiple sources, ensuring robustness in predictive modeling. These algorithms are particularly suited for structured data and multi-omics data integration, offering high accuracy and stability (21).

Large Language Models (LLM) in axSpA research: While machine learning has dominated axSpA research, large language models (LLMs) like GPT-4 and BERT are emerging as powerful tools in medical informatics. LLMs are particularly useful in natural language processing (NLP) tasks, such as extracting valuable insights from clinical records, medical literature, and patient narratives. These models facilitate the identification of disease patterns, biomarkers, and therapeutic targets by analyzing unstructured text data, offering an innovative approach to advancing early diagnosis and personalized treatment.

#### 2.5.2 Data integration and disease modeling

AI’s role extends to integrating multi-omics data—genomics, transcriptomics, proteomics, metabolomics, and microbiomics—enabling a more holistic understanding of axSpA pathophysiology. AI algorithms, such as autoencoders and factorization methods like MOFA+, allow researchers to uncover latent factors that influence disease progression, helping identify disease subtypes and novel therapeutic targets.

In the context of disease modeling, AI aids in developing dynamic models that predict disease progression and therapeutic responses, providing a personalized approach to axSpA management. Despite the progress, the scalability and clinical validation of these models remain challenges that require rigorous external validation through independent datasets and clinical trials.

#### 2.5.3 Challenges in AI implementation

While AI shows significant promise in improving diagnostic precision and treatment prediction, challenges remain in the scalability and clinical applicability of these models. Future research should focus on verifying AI models in diverse, multi-center clinical studies to ensure broad applicability across different patient populations. Additionally, addressing issues related to data heterogeneity, model transparency, and interpretability will be critical to fully integrate AI into clinical practice and provide trustworthy decision support.

### 2.6 Result validation and clinical application

The final stage of the pipeline involves rigorous functional and clinical validation of candidate biomarkers and therapeutic targets:

Functional validation: Includes *in vitro* assays and *in vivo* animal models to test biological relevance.

Clinical cohort validation: Evaluates biomarker performance across diverse patient populations.

Translation to clinical tools: Successful candidates may be developed into diagnostic assays or therapeutic strategies.

Through this translational pipeline, multi-omics research can generate actionable insights that improve early diagnosis, prognostic accuracy, and tailored therapy, thereby enhancing clinical outcomes and quality of life for axSpA patients.

## 3 Applications of omics technologies in axial spondyloarthritis

### 3.1 Genomic research and findings

Genomic technologies, particularly genome-wide association studies (GWAS), have played a pivotal role in exploring the genetic susceptibility to axSpA (22, 23). GWAS has identified several genetic factors associated with axSpA, with the HLA-B27 gene being the most well-known genetic marker (24). However, due to variations in the sensitivity and specificity of HLA-B27 across different ethnic groups, its universal applicability is limited (25, 26). To overcome this, researchers have employed polygenic risk scores (PRS), combining HLA-B27 with other related genes such as ERAP1 and IL23R, thereby significantly improving axSpA diagnostic accuracy (27). Additionally, polymorphisms in genes like STAT3 and TNFRSF1A have been found to be closely related to immune responses, providing new insights into the immune mechanisms of axSpA (28). Through genomic research, researchers not only identify new genetic markers but also offer theoretical support for personalized treatment, driving the development of targeted therapeutic strategies.

### 3.2 Transcriptomic research and findings

Transcriptomic technologies, especially RNA sequencing, have revealed significant differential expression of immune-related genes in axSpA patients, particularly pro-inflammatory cytokines such as TNF-α, IL-17, and IL-23, which play central roles in the immune response of axSpA (29). In conjunction with epigenetic studies, it has been found that hypomethylation of the IL-17A gene promoter region promotes excessive expression of IL-17A, providing new theoretical support for IL-17-targeted therapies (30, 31). Analysis of multiple datasets has also identified several key genes, such as ACSL1, SLC40A1, GZMM, TRIB1, and XBP1, which are closely associated with immune infiltration and disease activity in axSpA. Notably, the SLC40A1 gene, by regulating iron metabolism and ferroptosis, may exacerbate inflammation and drive axSpA progression (32). Furthermore, circRNA expression profiling in peripheral blood mononuclear cells has identified hsa_circRNA_001544 and hsa_circRNA_102532 as potential molecular biomarkers for axSpA, with hsa_circRNA_012732 potentially reflecting disease activity (33). By integrating transcriptomic and epigenetic analyses, these studies offer new biomarkers and therapeutic targets for precise diagnosis and personalized treatment of axSpA, laying the foundation for the development of immune-targeted therapies.

In the context of the vast amount of available ‘omics’ data, genomics and transcriptomics are currently considered the most crucial. HLA-B27 remains one of the most significant genetic markers for axSpA, and the integration of polygenic risk scores (PRS) provides additional predictive power. In terms of transcriptomics, the TNF-α and IL-17 signaling pathways play pivotal roles in the pathogenesis of axSpA and have a substantial impact on the development of targeted therapies.

### 3.3 The role of proteomics and metabolomics

Proteomics and metabolomics have provided critical information for understanding the pathological mechanisms of axSpA, particularly in identifying key proteins and metabolic products associated with disease progression (34). Mass spectrometry analyses have revealed widespread lipid metabolism dysregulation in axSpA patients, particularly the increased levels of phospholipids and sphingolipids, which are closely linked to the activation of the NF-κB signaling pathway (35). Changes in lipid metabolism may serve as a key driver of the inflammatory response in axSpA (36, 37). Metabolomic technologies have also uncovered metabolic abnormalities in axSpA patients, particularly enhanced glycolysis and decreased levels of short-chain fatty acids (38–40). These metabolic changes are significantly intertwined with immune responses, indicating that metabolic reprogramming plays a crucial role in the inflammatory process of axSpA. The study of metabolomics provides a new perspective on the mechanisms of axSpA and offers theoretical support for therapeutic strategies targeting metabolic regulation.

### 3.4 Breakthroughs in microbiomics

Research in gut microbiomics has offered new insights into axSpA. Studies have shown that the gut microbiota of axSpA patients exhibit significant structural differences, with some bacterial epitopes resembling the HLA-B27 gene, potentially inducing immune responses through molecular mimicry (41, 42). Metagenomic analyses have revealed that changes in the gut microbiome structure are closely associated with immune responses in axSpA. Notably, following treatment with TNF-α inhibitors (TNFi), the restoration of the gut microbiome reflects changes in immune responses, suggesting that the microbiome plays a key role in immune regulation in axSpA (17). These findings indicate that the gut microbiome may contribute to the pathogenesis of axSpA by influencing immune responses and autoimmune mechanisms, providing new clues for the potential role of the microbiome in axSpA treatment.

### 3.5 An integrated immunological model of axSpA pathogenesis

**Figure 2:**
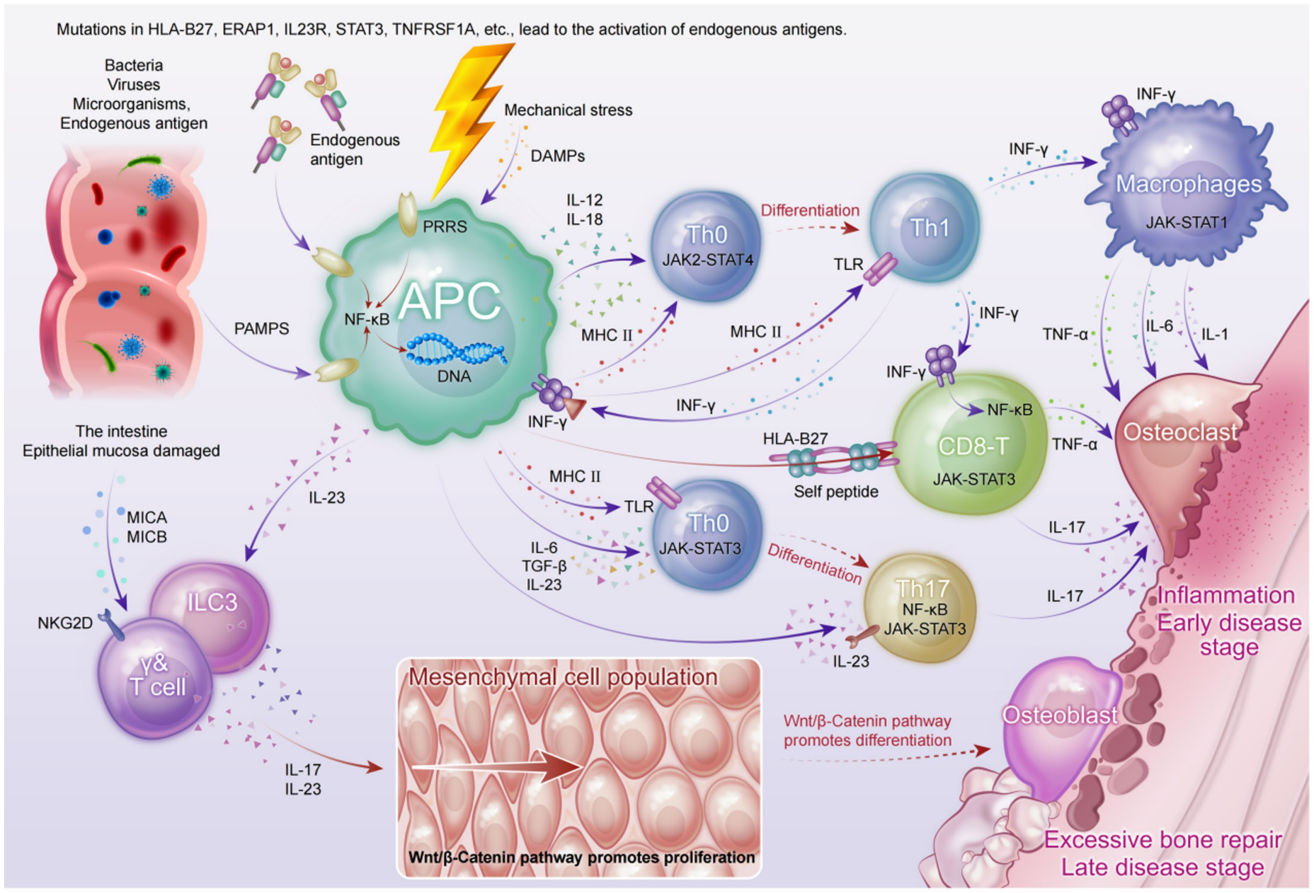
Integrated immunopathogenic model of axial spondyloarthritis.

Although the etiology of axSpA has not been fully elucidated, recent multi-omics studies have provided multi-layered evidence suggesting that its pathogenesis involves a complex interplay among genetic susceptibility, gut microbiota dysbiosis, immune dysregulation, and mechanical stress. Based on current findings from basic research, we have constructed an integrated immunological model of axSpA (see Figure 2) to better illustrate its complex pathological mechanisms.

At the genetic level, susceptibility genes—particularly HLA-B27—may contribute to aberrant antigen presentation, thereby inducing CD8^+^ T cell-mediated IFN-γ responses and activating canonical inflammatory signaling pathways such as JAK-STAT3 and NF-κB (43). This activation leads to the sustained production of pro-inflammatory cytokines including TNF-α, IL-6, and IL-1, establishing a chronic inflammatory milieu.

From a microbial perspective, gut dysbiosis in axSpA patients permits pathogen-associated molecular patterns (PAMPs) to traverse the impaired mucosal barrier, triggering pattern recognition receptors (PRRs) on dendritic cells and macrophages (44). This, in turn, promotes the secretion of IL-23 and IL-12, facilitating the differentiation of Th17 and Th1 cells. Activated Th17 cells and innate lymphoid cells type 3 (ILC3s) secrete large amounts of IL-17 in response to IL-23 stimulation, a pivotal cytokine in both enthesitis and bone remodeling (18).

Concurrently, mechanical stress at entheses induces the release of damage-associated molecular patterns (DAMPs), which recruit and activate γδ T cells and neutrophils. These cells further amplify the IL-17- and TNF-dominated inflammatory cascade, promoting bone resorption and tissue destruction (45).

As the disease progresses to later stages, chronic inflammation persistently activates the Wnt/β-catenin signaling pathway, aberrantly enhancing osteoblast activity and mesenchymal stem cell proliferation. This results in pathological new bone formation and the development of syndesmophytes (46). This inflammation-to-bone-remodeling transition elucidates the molecular basis of axSpA progression from bone erosion to ankylosis.

Although this model captures the major pathological pathways underlying articular manifestations in axSpA, it remains insufficient in explaining systemic features such as acute anterior uveitis (AAU). Future studies should leverage high-resolution technologies, including spatial transcriptomics and single-cell omics, to further unravel the dynamics of tissue microenvironments and immune cell lineages.

This schematic illustrates the interplay of genetic predisposition, microbial dysbiosis, immune dysregulation, and mechanical stress in the pathogenesis of axSpA. Genetic variants—particularly HLA-B27—contribute to aberrant antigen presentation and activation of CD8^+^ T cells, initiating IFN-γ production and downstream NF-κB and JAK-STAT3 signaling. Gut barrier dysfunction permits PAMPs to activate antigen-presenting cells (APCs), leading to IL-12 and IL-23 secretion and subsequent differentiation of Th1 and Th17 cells. Innate lymphoid cells (ILC3s) and γδ T cells further amplify IL-17–mediated inflammation in response to IL-23. Mechanical stress induces DAMP release, enhancing recruitment of pro-inflammatory immune cells. Chronic inflammation activates Wnt/β-catenin signaling, driving mesenchymal proliferation and osteoblast differentiation, culminating in pathological new bone formation and syndesmophyte development.

## 4 Applications of systems biology and multi-omics integration in axSpA research

Systems biology and multi-omics integration offer a novel perspective for axSpA research, particularly in elucidating the interactions between disease mechanisms and signaling pathways (see Figure 2). By integrating multi-omics data, systems biology not only deepens our understanding of the pathology of axSpA but also provides critical support for precision medicine (22).

### 4.1 Key network modeling and molecular mechanism analysis

Systems biology constructs protein–protein interaction (PPI) networks to identify core molecules and their signaling pathways in axSpA. Studies have shown that the NF-κB, JAK/STAT, and IL-23/IL-17 pathways play central roles in the immune regulation and inflammatory responses in axSpA (47–49). By combining proteomic and transcriptomic data, researchers have identified key genes such as IL-23R, ERAP1, and HLA-B27, which play significant roles in the pathogenesis of axSpA and have driven the development of targeted drugs and therapeutic strategies (50–52).

### 4.2 Dynamic network modeling and personalized treatment

Dynamic network modeling, which incorporates time-series data, offers valuable insights into the progression of axSpA, from early immune responses to the late development of bone lesions. This approach helps clarify the biological mechanisms driving the disease at different stages, particularly in the context of immune inflammation and bone remodeling. Research has shown that in the early stages of axSpA, the IL-23/IL-17 pathway plays a predominant role in initiating inflammation by promoting the proliferation and differentiation of Th17 cells. As the disease progresses, the Wnt signaling pathway becomes a key driver, regulating bone metabolism and contributing to the formation of bone lesions. Wnt activation exacerbates bone hypertrophy, which is critical in the later stages of disease progression (46, 53). These findings underscore the complex interactions between immune and skeletal systems that are central to axSpA.

Additionally, the IL-23/IL-17 signaling pathway interacts with the NF-κB pathway, enhancing the inflammatory response and simultaneously activating the Wnt pathway. This molecular cascade strengthens the link between immune inflammation and bone remodeling, offering a clear biological rationale for intervention at multiple disease stages (54–57). Based on dynamic network modeling, personalized treatment strategies for axSpA can be tailored to the patient’s stage of disease. In the early stage, targeting IL-17 with inhibitors like Secukinumab can effectively control the inflammatory response, potentially halting disease progression. In contrast, in the later stage, treatments focused on modulating bone metabolism have shown clinical promise in reducing bone lesions and preventing further joint fusion, highlighting their clinical relevance for improving patient outcomes and quality of life (58, 59).

## 5 Technological innovation and future development

With continuous advances in science and technology, research and treatment methods for axSpA are undergoing profound changes. From multi-omics integration to the application of AI, technological innovations are paving new pathways for precision medicine in axSpA (60).

### 5.1 Integration of single-cell omics and precision medicine

The rapid development of single-cell omics technologies offers new opportunities for precision medicine (61, 62). This technology allows for the precise identification of functional differences among various cell populations within the immune system of axSpA patients, revealing their specific roles in immune responses (63). It provides accurate immune targets for personalized treatment, especially in immunosuppressive therapies, enabling more targeted treatment plans based on the patient’s immune phenotype. Single-cell RNA sequencing has enabled in-depth analysis of dynamic changes in immune cells in axSpA patients, revealing the pivotal roles of Th17 and Treg cells in the immune response of axSpA (64). In the future, single-cell omics may be integrated with other omics technologies to provide more precise disease subtyping, helping physicians select the best treatment strategies for axSpA patients (65).

### 5.2 The prospects of spatial omics

Spatial omics technologies, particularly spatial transcriptomics, allow for precise localization of gene expression within tissue slices and enable the observation of spatial distribution and interactions between different cell types (66, 67). In axSpA research, spatial omics offers an opportunity to deeply understand changes in the microenvironment of bone and joint tissues (68, 69). This technology enables the exploration of interactions between immune cells and bone-metabolism cells in the bone and joint inflammation regions of axSpA, shedding light on their critical roles in disease pathogenesis. Specifically, in the study of bone hypertrophy and bone remodeling, spatial omics can accurately depict the spatial localization of different immune and bone cells, further uncovering their roles in disease (70). Spatial omics not only provides fundamental insights into disease mechanisms but also offers new biomarkers for clinical applications, driving personalized diagnosis and treatment of axSpA.

### 5.3 The deepening application of AI and data mining technologies

AI and machine learning have become indispensable tools in multi-omics data analysis for axSpA research (71). AI’s ability to identify disease patterns, predict therapeutic responses, and uncover novel biomarkers is transforming how we approach diagnosis and treatment.

#### 5.3.1 AI in data analysis and integration

AI for multi-omics integration: One of the most critical applications of AI in axSpA research is its ability to integrate multi-omics data from different layers—such as genomics, proteomics, and metabolomics. Machine learning algorithms enhance data integration by identifying hidden correlations and providing a more comprehensive view of the disease mechanisms (11). For example, AI models like XGBoost are being explored for predicting treatment responses based on multi-omics profiles, offering more personalized and effective treatment strategies.

Biomarker discovery and disease subtype classification: Unsupervised learning algorithms have the ability to discover previously unknown disease subtypes by analyzing complex datasets. These AI-driven models help identify specific biomarkers related to different disease stages and therapeutic responses, improving diagnostic precision. For example, MOFA+ has been used to integrate transcriptomics and proteomics data, identifying hidden factors that influence disease progression and treatment outcomes.

#### 5.3.2 AI in precision medicine

AI technologies contribute significantly to precision medicine by providing models that predict disease trajectories and patient-specific treatment responses. By leveraging multi-omics data, AI enhances the identification of molecular biomarkers and disease networks, helping to define personalized treatment regimens for axSpA (72). For instance, AI-driven predictive models can forecast patient responses to therapies like TNF-α inhibitors or IL-17 inhibitors, guiding clinicians in making more informed treatment decisions.

#### 5.3.3 Challenges and future prospects

Despite AI’s transformative potential, there are several challenges that must be addressed for widespread clinical adoption. Key issues include data privacy, model interpretability, and data validation. AI models in axSpA must undergo rigorous validation in independent datasets and clinical trials to confirm their reliability and applicability in real-world settings. Furthermore, addressing the biases inherent in AI algorithms and ensuring that models are transparent and explainable will be crucial to gaining clinical trust and ensuring equitable patient care.

The future of AI in axSpA lies in its ability to assist in clinical decision-making by integrating clinical, genomic, and treatment response data. By continuing to enhance AI’s analytical capabilities, the integration of multi-omics data will play a pivotal role in realizing personalized and effective treatments for axSpA.

### 5.4 New targeted therapies and immunomodulation strategies

The field of targeted therapy for axSpA is rapidly advancing, especially in immunomodulatory treatments. With a deeper understanding of the pathogenesis of axSpA, new targeted treatment strategies and immunomodulatory methods are gradually entering clinical practice. Currently, TNF-α inhibitors, IL-17 inhibitors, and JAK inhibitors have shown significant clinical effects in the treatment of axSpA, and more refined targeted drugs may emerge in the future (73–75). Targeted drugs that focus on different immune pathways will make axSpA treatment more personalized. Researchers are exploring immunotolerance strategies (e.g., antibody-dependent cytotoxicity) to modulate immune responses, thereby reducing abnormal activation of the immune system in axSpA patients, alleviating symptoms, and improving treatment efficacy. In the future, drug development will increasingly focus on precisely targeting specific molecules and cell subpopulations to enhance efficacy and reduce side effects. Drug development will not only rely on basic research but also integrate patients’ genomic and phenotypic information to create personalized treatment plans.

### 5.5 Clinical translation challenges in multi-omics integration

Despite significant progress in understanding axSpA through multi-omics technologies, clinical application still faces several challenges. These include data heterogeneity, the lack of standardized multi-omics integration protocols, and insufficient clinical validation of identified biomarkers and therapeutic targets (11). To overcome these challenges, several strategies should be considered:

Development of standardized methods for data preprocessing, integration, and interpretation: These methods will enhance the comparability of findings across different studies, making multi-omics data more reliable for clinical decision-making (76, 77).

Large-scale, multi-center clinical studies: To validate the clinical relevance of biomarkers and therapeutic targets identified through multi-omics integration, large-scale, multi-center studies are crucial. These studies will provide the necessary evidence to confirm the robustness and applicability of findings across diverse patient populations.

Collaborative data coordination and seamless integration: Coordinating data from multiple sources is vital for the seamless integration of clinical data, omics data, and patient metadata. This will mitigate the challenges posed by data heterogeneity and enable more accurate interpretation (78).

AI for data integration and analysis: AI, especially machine learning and deep learning, provides powerful tools for handling large, heterogeneous datasets. AI can optimize multi-omics data integration, identify meaningful patterns, and predict clinical outcomes more accurately, addressing issues such as data inconsistencies and missing information (79, 80). However, the interpretability, potential biases, and data privacy concerns of AI models must be carefully considered to ensure fairness and transparency in clinical applications.

Rigorous clinical validation: Clinical validation is essential for any potential biomarker or therapeutic target used in patient care. Structured validation processes, including *in vitro* testing, animal models, and extensive clinical trials, are crucial for confirming the results of multi-omics studies and ensuring their practical applicability.

By focusing on these strategies, multi-omics research can advance toward clinical translation, paving the way for more precise and effective treatments for axSpA.

## 6 Conclusion

axSpA is a complex immune-mediated inflammatory disease influenced by genetic, immune, and environmental factors. Although significant progress has been made in axSpA treatment, challenges remain in early diagnosis, personalized care, and long-term management. The rapid development of multi-omics technologies has provided new insights into disease mechanisms, and the integration of these approaches is driving the shift toward precision medicine. By integrating multi-omics data and utilizing AI for analysis, researchers can more accurately identify biomarkers and immune pathways, enabling the development of personalized treatment strategies. These advancements not only deepen our understanding of the pathophysiology of axSpA but also lay a solid foundation for the development of targeted therapies and optimized treatment plans. However, challenges such as data heterogeneity, lack of standardization, and difficulties in clinical translation continue to hinder the widespread implementation of these technologies. Overcoming these obstacles will require interdisciplinary collaboration, data sharing, and further advancements in AI technologies. As emerging tools such as single-cell omics and spatial omics continue to evolve, the integration of multi-omics with precision medicine will provide more effective and personalized treatments for axSpA patients, significantly improving their quality of life.

In summary, multi-omics research and precision medicine in axSpA hold great potential. Ongoing technological innovation and interdisciplinary collaboration will drive the clinical application of precision medicine in axSpA, optimizing diagnosis and treatment outcomes while improving long-term patient prognosis.

## Glossary

axSpA: Axial spondyloarthritis
AI: Artificial intelligence
GWAS: Genome-wide association study
PRS: Polygenic risk scores
HLA-B27: Human leukocyte antigen B27
IL: Interleukin
TNF-α: Tumor necrosis factor-alpha
TNFi: Tumor necrosis factor inhibitors
JAK: Janus kinase
STAT: Signal transducer and activator of transcription
ERAP1: Endoplasmic reticulum aminopeptidase 1
NF-κB: Nuclear factor kappa B
PPI: Protein–protein interaction
Th17: T helper 17 cells
Treg: Regulatory T cells
DKK-1: Dickkopf-related protein 1
Wnt: Wingless/integrated signaling pathway
LC–MS: Liquid chromatography-mass spectrometry
GC–MS: Gas chromatography–mass spectrometry
RNA-seq: RNA sequencing
scRNA-seq: Single-cell RNA sequencing
APCs: Antigen-presenting cells
PAMPs: Pathogen-associated molecular patterns
DAMPs: Damage-associated molecular patterns
IFN-γ: Interferon gamma
Th1: T-helper 1
CD8+ T cells: Cluster of differentiation 8 positive T cells
JAK-STAT: Janus kinase-signal transducer and activator of transcription
ILC3: Innate lymphoid cells type 3
γδT cells: Gamma delta T cells
AAU: Acute anterior uveitis
Wnt/β-catenin: Wnt/β-catenin signaling pathway

## References

[1] Generali E Bose T Selmi C Voncken JW Damoiseaux J. Nature versus nurture in the spectrum of rheumatic diseases: classification of spondyloarthritis as autoimmune or autoinflammatory. Autoimmun Rev. (2018) 17:935–41. doi: 10.1016/j.autrev.2018.04.002, PMID: 30005857

[2] Zhu W He X Cheng K Zhang L Chen D Wang X . Ankylosing spondylitis: etiology, pathogenesis, and treatments. Bone Res. (2019) 7:22. doi: 10.1038/s41413-019-0057-8, PMID: 31666997 PMC6804882

[3] Rudwaleit M Landewe R Sieper J. Ankylosing spondylitis and axial spondyloarthritis. N Engl J Med. (2016) 375:1302–3. doi: 10.1056/NEJMc1609622, PMID: 27682052

[4] Bittar M Deodhar A. Axial spondyloarthritis: a review. JAMA. (2024) 333:408–20. doi: 10.1001/jama.2024.20917 39630439

[5] Navarro-Compan V Sepriano A Capelusnik D Baraliakos X. Axial spondyloarthritis. Lancet. (2025) 405:159–72. doi: 10.1016/S0140-6736(24)02263-3, PMID: 39798984

[6] Derakhshan MH Pathak H Cook D Dickinson S Siebert S Gaffney K. Services for spondyloarthritis: a survey of patients and rheumatologists. Rheumatology (Oxford). (2018) 57:987–96. doi: 10.1093/rheumatology/kex518, PMID: 29529295

[7] Stolwijk C Essers I van Tubergen A Boonen A Bazelier MT de Bruin ML . The epidemiology of extra-articular manifestations in ankylosing spondylitis: a population-based matched cohort study. Ann Rheum Dis. (2015) 74:1373–8. doi: 10.1136/annrheumdis-2014-205253, PMID: 24658834

[8] He X Liu X Zuo F Shi H Jing J. Artificial intelligence-based multi-omics analysis fuels cancer precision medicine. Semin Cancer Biol. (2023) 88:187–200. doi: 10.1016/j.semcancer.2022.12.009, PMID: 36596352

[9] Thomas GP Brown MA. Genetics and genomics of ankylosing spondylitis. Immunol Rev. (2010) 233:162–80. doi: 10.1111/j.0105-2896.2009.00852.x, PMID: 20192999

[10] Chen C Wang J Pan D Wang X Xu Y Yan J . Applications of multi-omics analysis in human diseases. MedComm (2020). (2023) 4:e315. doi: 10.1002/mco2.315 37533767 PMC10390758

[11] Zitnik M Nguyen F Wang B Leskovec J Goldenberg A Hoffman MM. Machine learning for integrating data in biology and medicine: principles, practice, and opportunities. Inf Fusion. (2019) 50:71–91. doi: 10.1016/j.inffus.2018.09.012, PMID: 30467459 PMC6242341

[12] Seyed Tabib NS Madgwick M Sudhakar P Verstockt B Korcsmaros T Vermeire S. Big data in IBD: big progress for clinical practice. Gut. (2020) 69:1520–32. doi: 10.1136/gutjnl-2019-320065, PMID: 32111636 PMC7398484

[13] Yuzbasioglu A Ozguc M. Biobanking: sample acquisition and quality assurance for 'omics' research. New Biotechnol. (2013) 30:339–42. doi: 10.1016/j.nbt.2012.11.016, PMID: 23183539

[14] Duan R Leo P Bradbury L Brown MA Thomas G. Gene expression profiling reveals a downregulation in immune-associated genes in patients with axSpA. Ann Rheum Dis. (2010) 69:1724–9. doi: 10.1136/ard.2009.111690, PMID: 19643760

[15] Wright C Edelmann M diGleria K Kollnberger S Kramer H McGowan S . Ankylosing spondylitis monocytes show upregulation of proteins involved in inflammation and the ubiquitin proteasome pathway. Ann Rheum Dis. (2009) 68:1626–32. doi: 10.1136/ard.2008.097204, PMID: 18952638

[16] Rizzo C Camarda F Donzella D La Barbera L Guggino G. Metabolomics: an emerging approach to understand pathogenesis and to assess diagnosis and response to treatment in spondyloarthritis. Cells. (2022) 11:549. doi: 10.3390/cells11030549, PMID: 35159358 PMC8834108

[17] Yin J Sternes PR Wang M Song J Morrison M Li T . Shotgun metagenomics reveals an enrichment of potentially cross-reactive bacterial epitopes in ankylosing spondylitis patients, as well as the effects of TNFi therapy upon microbiome composition. Ann Rheum Dis. (2020) 79:132–40. doi: 10.1136/annrheumdis-2019-215763, PMID: 31662318

[18] Gill T Asquith M Rosenbaum JT Colbert RA. The intestinal microbiome in spondyloarthritis. Curr Opin Rheumatol. (2015) 27:319–25. doi: 10.1097/BOR.0000000000000187, PMID: 26002022 PMC4489849

[19] Goh WWB Wang W Wong L. Why batch effects matter in omics data, and how to avoid them. Trends Biotechnol. (2017) 35:498–507. doi: 10.1016/j.tibtech.2017.02.012, PMID: 28351613

[20] Tong L Shi W Isgut M Zhong Y Lais P Gloster L . Integrating multi-omics data with EHR for precision medicine using advanced artificial intelligence. IEEE Rev Biomed Eng. (2024) 17:80–97. doi: 10.1109/RBME.2023.3324264, PMID: 37824325

[21] Zhang W Li Y Shao P du Y Zhao K Zhan J . Association of weight-adjusted waist index and body mass index with chronic low back pain in American adults: a retrospective cohort study and predictive model development based on machine learning algorithms (NHANES 2009-2010). Front Public Health. (2025) 13:1617732. doi: 10.3389/fpubh.2025.1617732, PMID: 40717955 PMC12289706

[22] Australo-Anglo-American Spondyloarthritis Consortium (TASC) Reveille JD Sims AM Danoy P Evans DM Leo P . Genome-wide association study of ankylosing spondylitis identifies non-MHC susceptibility loci. Nat Genet. (2010) 42:123–7. doi: 10.1038/ng.513 20062062 PMC3224997

[23] Lin Z Bei J-X Shen M Li Q Liao Z Zhang Y . A genome-wide association study in Han Chinese identifies new susceptibility loci for ankylosing spondylitis. Nat Genet. (2011) 44:73–7. doi: 10.1038/ng.1005, PMID: 22138694

[24] Brown MA Kenna T Wordsworth BP. Genetics of ankylosing spondylitis--insights into pathogenesis. Nat Rev Rheumatol. (2016) 12:81–91. doi: 10.1038/nrrheum.2015.133, PMID: 26439405

[25] Wu X Wang G Zhang L Xu H. Genetics of ankylosing spondylitis-focusing on the ethnic difference between East Asia and Europe. Front Genet. (2021) 12:671682. doi: 10.3389/fgene.2021.671682, PMID: 34194471 PMC8236852

[26] Wang G Kim TH Li Z Cortes A Kim K Bang SY . MHC associations of ankylosing spondylitis in east Asians are complex and involve non-HLA-B27 HLA contributions. Arthritis Res Ther. (2020) 22:74. doi: 10.1186/s13075-020-02148-5, PMID: 32272966 PMC7146985

[27] Li Z Wu X Leo PJ de Guzman E Akkoc N Breban M . Polygenic risk scores have high diagnostic capacity in ankylosing spondylitis. Ann Rheum Dis. (2021) 80:1168–74. doi: 10.1136/annrheumdis-2020-219446, PMID: 34161253 PMC8364478

[28] Davidson SI Liu Y Danoy PA Wu X Thomas GP Jiang L . Association of STAT3 and TNFRSF1A with ankylosing spondylitis in Han Chinese. Ann Rheum Dis. (2011) 70:289–92. doi: 10.1136/ard.2010.133322, PMID: 21068102

[29] Gaffen SL Jain R Garg AV Cua DJ. The IL-23-IL-17 immune axis: from mechanisms to therapeutic testing. Nat Rev Immunol. (2014) 14:585–600. doi: 10.1038/nri3707, PMID: 25145755 PMC4281037

[30] Roberts AR Vecellio M Chen L Ridley A Cortes A Knight JC . An ankylosing spondylitis-associated genetic variant in the IL23R-IL12RB2 intergenic region modulates enhancer activity and is associated with increased Th1-cell differentiation. Ann Rheum Dis. (2016) 75:2150–6. doi: 10.1136/annrheumdis-2015-208640, PMID: 26916345 PMC5136719

[31] Yang BH Floess S Hagemann S Deyneko IV Groebe L Pezoldt J . Development of a unique epigenetic signature during *in vivo* Th17 differentiation. Nucleic Acids Res. (2015) 43:1537–48. doi: 10.1093/nar/gkv014, PMID: 25593324 PMC4330377

[32] Ni Y Zhong L Li Y Zhang Z Ming B Qing Y . Exploration of molecular biomarkers in ankylosing spondylitis transcriptomics. Front Immunol. (2024) 15:1480492. doi: 10.3389/fimmu.2024.1480492, PMID: 39759509 PMC11695275

[33] Tang YP Zhang QB Dai F Liao X Dong ZR Yi T . Circular RNAs in peripheral blood mononuclear cells from ankylosing spondylitis. Chin Med J. (2021) 134:2573–82. doi: 10.1097/CM9.0000000000001815, PMID: 34670246 PMC8577680

[34] Ondrejcakova L Gregova M Bubova K Senolt L Pavelka K. Serum biomarkers and their relationship to axial spondyloarthritis associated with inflammatory bowel diseases. Autoimmun Rev. (2024) 23:103512. doi: 10.1016/j.autrev.2023.103512, PMID: 38168574

[35] Sundstrom B Johansson G Kokkonen H Cederholm T Wallberg-Jonsson S. Plasma phospholipid fatty acid content is related to disease activity in ankylosing spondylitis. J Rheumatol. (2012) 39:327–33. doi: 10.3899/jrheum.110575, PMID: 22174215

[36] Xu WD Yang XY Li DH Zheng KD Qiu PC Zhang W . Up-regulation of fatty acid oxidation in the ligament as a contributing factor of ankylosing spondylitis: a comparative proteomic study. J Proteome. (2015) 113:57–72. doi: 10.1016/j.jprot.2014.09.014, PMID: 25281561

[37] Li Z Gu W Wang Y Qin B Ji W Wang Z . Untargeted lipidomics reveals characteristic biomarkers in patients with ankylosing spondylitis disease. Biomedicine. (2022) 11:47. doi: 10.3390/biomedicines11010047, PMID: 36672555 PMC9855684

[38] Guo Y Wei S Yin M Cao D Li Y Wen C . Gas chromatography-mass spectrometry reveals stage-specific metabolic signatures of ankylosing spondylitis. Meta. (2023) 13:1058. doi: 10.3390/metabo13101058, PMID: 37887383 PMC10608640

[39] Gao P Lu C Zhang F Sang P Yang D Li X . Integrated GC-MS and LC-MS plasma metabonomics analysis of ankylosing spondylitis. Analyst. (2008) 133:1214–20. doi: 10.1039/b807369d, PMID: 18709197

[40] Ou J Xiao M Huang Y Tu L Chen Z Cao S . Serum metabolomics signatures associated with ankylosing spondylitis and TNF inhibitor therapy. Front Immunol. (2021) 12:630791. doi: 10.3389/fimmu.2021.630791, PMID: 33679777 PMC7933516

[41] Asquith M Sternes PR Costello ME Karstens L Diamond S Martin TM . HLA alleles associated with risk of ankylosing spondylitis and rheumatoid arthritis influence the gut microbiome. Arthritis Rheumatol. (2019) 71:1642–50. doi: 10.1002/art.40917, PMID: 31038287

[42] Xu H Yin J. HLA risk alleles and gut microbiome in ankylosing spondylitis and rheumatoid arthritis. Best Pract Res Clin Rheumatol. (2019) 33:101499. doi: 10.1016/j.berh.2020.101499, PMID: 32279929

[43] Bowness P. Hla-B27. Annu Rev Immunol. (2015) 33:29–48. doi: 10.1146/annurev-immunol-032414-112110, PMID: 25861975

[44] Cua DJ Sherlock JP. Autoimmunity's collateral damage: gut microbiota strikes 'back. Nat Med. (2011) 17:1055–6. doi: 10.1038/nm0911-1055, PMID: 21900923

[45] Schett G Lories RJ D'Agostino MA Elewaut D Kirkham B Soriano ER . Enthesitis: from pathophysiology to treatment. Nat Rev Rheumatol. (2017) 13:731–41. doi: 10.1038/nrrheum.2017.188, PMID: 29158573

[46] Li X Wang J Zhan Z Li S Zheng Z Wang T . Inflammation intensity-dependent expression of Osteoinductive Wnt proteins is critical for ectopic new bone formation in ankylosing spondylitis. Arthritis Rheumatol. (2018) 70:1056–70. doi: 10.1002/art.40468, PMID: 29481736

[47] Hwang M Assassi S Zheng J Castillo J Chavez R Vanarsa K . Quantitative proteomic screening uncovers candidate diagnostic and monitoring serum biomarkers of ankylosing spondylitis. Arthritis Res Ther. (2023) 25:57. doi: 10.1186/s13075-023-03044-4, PMID: 37041650 PMC10088143

[48] Klavdianou K Tsiami S Baraliakos X. New developments in ankylosing spondylitis-status in 2021. Rheumatology (Oxford). (2021) 60:vi29-vi37. doi: 10.1093/rheumatology/keab523, PMID: 34951921 PMC8709566

[49] Guo Q Jin Y Chen X Ye X Shen X Lin M . NF-kappaB in biology and targeted therapy: new insights and translational implications. Signal Transduct Target Ther. (2024) 9:53. doi: 10.1038/s41392-024-01757-9 38433280 PMC10910037

[50] Haroon N Inman RD. ERAP1 and the return of the UPR in ankylosing spondylitis. Nat Rev Rheumatol. (2023) 19:134–5. doi: 10.1038/s41584-023-00910-y, PMID: 36725927

[51] Evans DM Spencer CCA Pointon JJ Su Z Harvey D Kochan G . Interaction between ERAP1 and HLA-B27 in ankylosing spondylitis implicates peptide handling in the mechanism for HLA-B27 in disease susceptibility. Nat Genet. (2011) 43:761–7. doi: 10.1038/ng.873, PMID: 21743469 PMC3640413

[52] Rueda B Orozco G Raya E Fernandez-Sueiro JL Mulero J Blanco FJ . The IL23R Arg381Gln non-synonymous polymorphism confers susceptibility to ankylosing spondylitis. Ann Rheum Dis. (2008) 67:1451–4. doi: 10.1136/ard.2007.080283, PMID: 18199597

[53] Gravallese EM Schett G. Effects of the IL-23-IL-17 pathway on bone in spondyloarthritis. Nat Rev Rheumatol. (2018) 14:631–40. doi: 10.1038/s41584-018-0091-8, PMID: 30266977

[54] Muranski P Borman ZA Kerkar SP Klebanoff CA Ji Y Sanchez-Perez L . Th17 cells are long lived and retain a stem cell-like molecular signature. Immunity. (2011) 35:972–85. doi: 10.1016/j.immuni.2011.09.019, PMID: 22177921 PMC3246082

[55] Chae WJ Bothwell ALM. Canonical and non-canonical Wnt signaling in immune cells. Trends Immunol. (2018) 39:830–47. doi: 10.1016/j.it.2018.08.006, PMID: 30213499 PMC7367500

[56] Daoussis D Andonopoulos AP Liossis SN. Wnt pathway and IL-17: novel regulators of joint remodeling in rheumatic diseases. Looking beyond the RANK-RANKL-OPG axis. Semin Arthritis Rheum. (2010) 39:369–83. doi: 10.1016/j.semarthrit.2008.10.008, PMID: 19095294

[57] Uderhardt S Diarra D Katzenbeisser J David JP Zwerina J Richards W . Blockade of Dickkopf (DKK)-1 induces fusion of sacroiliac joints. Ann Rheum Dis. (2010) 69:592–7. doi: 10.1136/ard.2008.102046, PMID: 19304568

[58] Baeten D Sieper J Braun J Baraliakos X Dougados M Emery P . Secukinumab, an interleukin-17A inhibitor, in ankylosing spondylitis. N Engl J Med. (2015) 373:2534–48. doi: 10.1056/NEJMoa1505066, PMID: 26699169

[59] Cairns AP Wright SA Taggart AJ Coward SM Wright GD. An open study of pulse pamidronate treatment in severe ankylosing spondylitis, and its effect on biochemical markers of bone turnover. Ann Rheum Dis. (2005) 64:338–9. doi: 10.1136/ard.2004.022871, PMID: 15096328 PMC1755338

[60] Belge Bilgin G Bilgin C Burkett BJ Orme JJ Childs DS Thorpe MP . Theranostics and artificial intelligence: new frontiers in personalized medicine. Theranostics. (2024) 14:2367–78. doi: 10.7150/thno.94788, PMID: 38646652 PMC11024845

[61] Zeng L Yang K Zhang T Zhu X Hao W Chen H . Research progress of single-cell transcriptome sequencing in autoimmune diseases and autoinflammatory disease: a review. J Autoimmun. (2022) 133:102919. doi: 10.1016/j.jaut.2022.102919, PMID: 36242821

[62] Vandereyken K Sifrim A Thienpont B Voet T. Methods and applications for single-cell and spatial multi-omics. Nat Rev Genet. (2023) 24:494–515. doi: 10.1038/s41576-023-00580-2, PMID: 36864178 PMC9979144

[63] Alber S Kumar S Liu J Huang ZM Paez D Hong J . Single cell transcriptome and surface epitope analysis of ankylosing spondylitis facilitates disease classification by machine learning. Front Immunol. (2022) 13:838636. doi: 10.3389/fimmu.2022.838636, PMID: 35634297 PMC9135966

[64] Yi K Jo S Song W Lee H‐I Kim H‐J Kang J‐H . Analysis of single-cell transcriptome and surface protein expression in ankylosing spondylitis identifies OX40-positive and glucocorticoid-induced tumor necrosis factor receptor-positive pathogenic Th17 cells. Arthritis Rheumatol. (2023) 75:1176–86. doi: 10.1002/art.42476, PMID: 36787119

[65] Ma A McDermaid A Xu J Chang Y Ma Q. Integrative methods and practical challenges for single-cell multi-omics. Trends Biotechnol. (2020) 38:1007–22. doi: 10.1016/j.tibtech.2020.02.013, PMID: 32818441 PMC7442857

[66] Bressan D Battistoni G Hannon GJ. The dawn of spatial omics. Science. (2023) 381:eabq4964. doi: 10.1126/science.abq4964, PMID: 37535749 PMC7614974

[67] Anderson AC Yanai I Yates LR Wang L Swarbrick A Sorger P . Spatial transcriptomics. Cancer Cell. (2022) 40:895–900. doi: 10.1016/j.ccell.2022.08.021, PMID: 36099884

[68] Akbar M MacDonald L Crowe LAN Carlberg K Kurowska-Stolarska M Ståhl PL . Single cell and spatial transcriptomics in human tendon disease indicate dysregulated immune homeostasis. Ann Rheum Dis. (2021) 80:1494–7. doi: 10.1136/annrheumdis-2021-220256, PMID: 34001518 PMC8522454

[69] Feng S Li J Tian J Lu S Zhao Y. Application of single-cell and spatial omics in musculoskeletal disorder research. Int J Mol Sci. (2023) 24:2271. doi: 10.3390/ijms24032271, PMID: 36768592 PMC9917071

[70] Kang H Strong AL Sun Y Guo L Juan C Bancroft AC . The HIF-1alpha/PLOD2 axis integrates extracellular matrix organization and cell metabolism leading to aberrant musculoskeletal repair. Bone Res. (2024) 12:17. doi: 10.1038/s41413-024-00320-0, PMID: 38472175 PMC10933265

[71] Matuchansky C. Deep medicine, artificial intelligence, and the practising clinician. Lancet. (2019) 394:736. doi: 10.1016/S0140-6736(19)31235-8, PMID: 31478500

[72] Wang R Dasgupta A Ward MM. Predicting probability of response to tumor necrosis factor inhibitors for individual patients with ankylosing spondylitis. JAMA Netw Open. (2022) 5:e222312. doi: 10.1001/jamanetworkopen.2022.2312, PMID: 35289857 PMC8924712

[73] Huang F Gu J Zhu P Bao C Xu J Xu H . Efficacy and safety of adalimumab in Chinese adults with active ankylosing spondylitis: results of a randomised, controlled trial. Ann Rheum Dis. (2014) 73:587–94. doi: 10.1136/annrheumdis-2012-202533, PMID: 23475983

[74] Baraliakos X Deodhar A van der Heijde D Magrey M Maksymowych WP Tomita T . Bimekizumab treatment in patients with active axial spondyloarthritis: 52-week efficacy and safety from the randomised parallel phase 3 BE MOBILE 1 and BE MOBILE 2 studies. Ann Rheum Dis. (2024) 83:1–15. doi: 10.1136/ard-2023-224803, PMID: 37793792

[75] Deodhar A Sliwinska-Stanczyk P Xu H Baraliakos X Gensler LS Fleishaker D . Tofacitinib for the treatment of ankylosing spondylitis: a phase III, randomised, double-blind, placebo-controlled study. Ann Rheum Dis. (2021) 80:1004–13. doi: 10.1136/annrheumdis-2020-219601, PMID: 33906853 PMC8292568

[76] Karczewski KJ Snyder MP. Integrative omics for health and disease. Nat Rev Genet. (2018) 19:299–310. doi: 10.1038/nrg.2018.4, PMID: 29479082 PMC5990367

[77] Ritchie MD Holzinger ER Li R Pendergrass SA Kim D. Methods of integrating data to uncover genotype-phenotype interactions. Nat Rev Genet. (2015) 16:85–97. doi: 10.1038/nrg3868, PMID: 25582081

[78] Zitnik M Zupan B. Jumping across biomedical contexts using compressive data fusion. Bioinformatics. (2016) 32:i90–i100. doi: 10.1093/bioinformatics/btw247, PMID: 27307649 PMC4908331

[79] Boehm KM Khosravi P Vanguri R Gao J Shah SP. Harnessing multimodal data integration to advance precision oncology. Nat Rev Cancer. (2022) 22:114–26. doi: 10.1038/s41568-021-00408-3, PMID: 34663944 PMC8810682

[80] Stafford IS Kellermann M Mossotto E Beattie RM MacArthur BD Ennis S. A systematic review of the applications of artificial intelligence and machine learning in autoimmune diseases. NPJ Digit Med. (2020) 3:30. doi: 10.1038/s41746-020-0229-3, PMID: 32195365 PMC7062883

